# Rapid heartbeat, but dry palms: reactions of heart rate and skin conductance levels to social rejection

**DOI:** 10.3389/fpsyg.2014.00956

**Published:** 2014-08-29

**Authors:** Benjamin Iffland, Lisa M. Sansen, Claudia Catani, Frank Neuner

**Affiliations:** ^1^Department of Psychology, Bielefeld UniversityBielefeld, Germany; ^2^Christoph-Dornier-Stiftung für Klinische PsychologieBielefeld, Germany

**Keywords:** Cyberball, social exclusion, autonomic arousal, physiological indices, heart rate, skin conductance

## Abstract

**Background:** Social rejection elicits negative mood, emotional distress, and neural activity in networks that are associated with physical pain. However, studies assessing physiological reactions to social rejection are rare and results of these studies were found to be ambiguous. Therefore, the present study aimed to examine and specify physiological effects of social rejection.

**Methods:** Participants (*n* = 50) were assigned to either a social exclusion or inclusion condition of a virtual ball-tossing game (Cyberball). Immediate and delayed physiological [skin conductance level (SCL) and heart rate] reactions were recorded. In addition, subjects reported levels of affect, emotional states, and fundamental needs.

**Results:** Subjects who were socially rejected showed increased heart rates. However, social rejection had no effect on subjects' SCLs. Both conditions showed heightened arousal on this measurement. Furthermore, psychological consequences of social rejection indicated the validity of the paradigm.

**Conclusions:** Our results reveal that social rejection evokes an immediate physiological reaction. Accelerated heart rates indicate that behavior activation rather than inhibition is associated with socially threatening events. In addition, results revealed gender-specific response patterns suggesting that sample characteristics such as differences in gender may account for ambiguous findings of physiological reactions to social rejection.

## Introduction

Experiences of social rejection and exclusion cause immediate distress and are associated with the development of psychosomatic problems, health risk factors, i.e., smoking, obesity, or high blood pressure, and a wide range of psychological disorders (Bell-Dolan et al., 1995; Reinherz et al., 2000; Deater-Deckard, 2001; Hock and Lutz, 2001; Nolan et al., 2003; Uchino, 2006). The immediate reactions to social rejection have been investigated in many studies using various experimental designs (Williams et al., 2000, 2002; Smith and Williams, 2004; Zadro et al., 2004; Gonsalkorale and Williams, 2007). In summary, social rejection generally elicits negative mood, emotional distress, and reduced feelings of belonging, self-esteem, and control (e.g., Williams et al., 2000; Leary et al., 2001; Buckley et al., 2004; Zadro et al., 2004; Gonsalkorale and Williams, 2007; Williams, 2007). At the same time, social exclusion activates neural networks that are associated with the processing of pain and distress (Eisenberger et al., 2003, 2007, 2011; Eisenberger and Lieberman, 2004; Somerville et al., 2006; Krill and Platek, 2009; Onoda et al., 2009, 2010; Yanagisawa et al., 2011a,b; DeWall et al., 2012; Kawamoto et al., 2012), in particular the dorsal anterior cingulated cortex (dACC) and the right ventrolateral prefrontal cortex (rVLPFC). On a hormonal level, several studies reported enhanced cortisol activity in response to rejection (Stroud et al., 2002; Blackhart et al., 2007; Zwolinski, 2008), which corresponds to the hypothesis that the cortisol-eliciting hypothalamic-pituitary-adrenocortical (HPA) stress axis is responsive to social threat (Erickson et al., 2003; Dickerson and Kemeny, 2004; Lovallo et al., 2012).

Although the central and hormonal stress reaction in response to rejection has been documented in many studies (Critchley et al., 2003; Nagai et al., 2004; Wong et al., 2007; Åhs et al., 2009; Lane et al., 2009), the corresponding peripheral physiological stress effects of social rejection on heart rate, skin conductance and other parameters of the autonomic nervous system (ANS) are still unclear. In one study, social rejection caused a transient slowing rather than an increase of heart rate in response to rejecting feedback of mock-peers in a social-judgment task (Gunther Moor et al., 2010). However, social rejection caused the opposite pattern, i.e., a sympathetic activation and a reduction of parasympathetic activity in other experiments documented by increased heart rate and respiratory sinus arrhythmia (RSA) withdrawal (Murray-Close, 2011) as well as increased skin conductance levels (SCL; Murray-Close, 2011; Shoulberg et al., 2011; Sijtsema et al., 2011; Kelly et al., 2012). These studies used the so-called Cyberball paradigm to simulate social rejection that showed reliable effects in eliciting feelings of social exclusion (Williams et al., 2000; Williams and Jarvis, 2006). In this paradigm, participants are told that they would be playing an Internet ball-tossing game with two or more co-players on the computer. However, these players are in fact simulated by the computer and, in the exclusion condition, programmed to throw the ball to the subject only once and to ignore the subject as the game continues. Using this paradigm, two studies reported a smaller decrease or an increase of skin conductance in the exclusion group compared to a control group that was not excluded in the game. Consistent with this finding, increased SCLs in excluded participants were reported by Murray-Close (2011). However, the majority of studies could not find any reliable physiological or hormonal stress response in the Cyberball game (Krimsky, 2010; Weik et al., 2010; Zöller et al., 2010; Shoulberg et al., 2011; Sijtsema et al., 2011; Iffland et al., 2014) or reported small gender-specific effects, i.e., cortisol responds in women, but not in men (Stroud et al., 2002; Weik et al., 2010). These findings indicate that the rejection experienced in the Cyberball paradigm may not be intense enough to elicit a full-blown stress response (Krimsky, 2010).

On the background of the ambiguity of previous findings of peripheral physiological reactions to social rejection, the present study aimed to examine and specify effects of social rejection on physiological outcomes. We applied the Cyberball game as one of the best established paradigms to induce feelings of social rejection. A prediction of the direction of the physiological response was not possible as both increases and decreases in bodily responses as measured by SCL and heart rate were plausible reactions to an episode of social rejection. As an improvement on previous research with the Cyberball game that used samples with predominantly female subjects (Gunther Moor et al., 2010; Zöller et al., 2010; Murray-Close, 2011; Shoulberg et al., 2011; Sijtsema et al., 2011; Kelly et al., 2012) and that did not provide a non-rejection condition for comparison (Murray-Close, 2011; Shoulberg et al., 2011; Sijtsema et al., 2011; Iffland et al., 2014), this study aimed for an equal sex ratio and implemented a social exclusion as well as an inclusion condition which were compared in a between subjects design. In addition, participants' need for belonging, self-esteem, control, and meaningful existence, as well as positive and negative affect, and emotional reactions were analyzed to control for psychological effects of the Cyberball game.

## Methods

### Participants

Participants were recruited through advertisements at the campus of Bielefeld University and were paid for their participation. Participants included 50 (25 female) individuals. Subjects currently enrolled at the faculty of psychology at Bielefeld University were rejected from participation as it was felt that they might be too suspicious of the experimental manipulation. The demographic characteristics of the sample are presented in Table 1.

**Table 1:** **Subject characteristics and mean values on the assessments (*n* = 50)**.

	**Total (*N* = 50)**	**Ostracism (*n* = 25)**	**Inclusion (*n* = 25)**	***p***
Age, *M* (*SD*, range)	24.04 (3.11, 18–29)	24.04 (3.18, 20–29)	24.04 (3.11, 18–29)	n.s.
Gender, *% female* (*n*)	50.0 (25)	52.0 (13)	48.0 (12)	n.s.^a^
Family status, *% single* (*n*)	74.0 (37)	76.0 (19)	72.0 (18)	n.s.^a^
Social phobia scale, *M* (*SD*)	12.70 (9.08)	13.32 (11.05)	12.08 (6.73)	n.s.
Social interaction anxiety scale, *M* (*SD*)	20.22 (12.13)	21.72 (14.13)	18.72 (9.82)	n.s.
Beck depression inventory, *M* (*SD*)	7.56 (4.59)	7.20 (4.91)	7.92 (4.33)	n.s.
Brief symptom inventory—global severity index, *M* (*SD*)	0.54 (0.45)	0.57 (0.54)	0.52 (0.34)	n.s.

a *Chi-Quadrat-Test*.

### Instruments

#### Manipulation checks and confounding factors

There were two manipulation checks to confirm participants' perception of their inclusionary status. Firstly, they were asked to estimate the percent of throws they had received (“Assuming that 33% of the time you would receive the ball if everyone received it equally, what percent of the throws did you receive?”). Secondly, they were asked to rate how much they felt excluded while playing the Cyberball game on a 9-point Likert scale ranging from 1 (very included) to 9 (very excluded). In addition, participants had the chance to write down their thoughts during the Cyberball game (Williams et al., 2000) and to comment on their thoughts. Furthermore, participants rated their imagination ability, vividness of imagination, subjective arousal during imagination, and familiarity to the situation on a 7-point Likert scale (“How well were you able to visualize the scene?” “How real did the scene you imagined seem?” “How vivid was your imagination?” “Have you been aroused while imagining the scene?” “Has the imagined scene been familiar to you?”).

#### Positive and negative affect

For the assessment of positive and negative affect in reaction to social exclusion the German version of the Positive and Negative Affect Schedule was used (PANAS; Watson et al., 1988; Krohne et al., 1996). The PANAS was developed to assess positive and negative affect measured on a five-point Likert scale ranging from 1 (very slightly) to 5 (extremely). It is intended to gain information on a participant's emotional state at the moment that the questionnaire is given. The two scales positive and negative affect consist of ten items each. The scales were shown to be largely uncorrelated (Watson et al., 1988). The German version showed good internal consistency (Cronbach's α > 0.84; Krohne et al., 1996).

#### Self-reported levels of needs

Levels of primary needs were assessed using a German translation of a questionnaire that has been used in previous cyberostracism research (Zadro et al., 2004). The questionnaire consists of 12 items assessing the four fundamental needs Belonging (e.g., “I felt like an outsider during the Cyberball game”), Self-Esteem (e.g., “During the Cyberball game, I felt good about myself”), Control (e.g., “I felt that I was able to throw the ball as often as I wanted during the game”), and Meaningful Existence (e.g., “I felt non-existent during the Cyberball game”). All items were rated on a 9-point Likert scale ranging from 1 (not at all) to 9 (very much so). In the present study, internal consistency of all subscales was acceptable (all Cronbach's α's > 0.72).

#### Emotional reactions

Participants were asked to rate intensity of seven emotional reactions (anxiety, sadness, anger, guilt, disgust, shame, happiness). Each emotional reaction was rated using a 7-point Likert scale ranging from 1 (very weak) to 7 (very strong).

#### Social anxiety symptoms

For the assessment of social phobia, the German version of the Social Phobia Scale/Social Interaction Anxiety Scale (SPS/SIAS; Heinrichs et al., 2002) was used. The SPS was developed to assess anxiety related specifically to social performance, whereas the SIAS was designed to measure anxiety related to general social interaction. Both, the SPS and the SIAS consist of 20 items using a five-point Likert scale that are rated from 0 (not at all) to 4 (extremely) indicating how characteristic or true the statements are for the respondent. On both scales total scores range from 0 to 80. Cut-off scores of 20 on the SPS and 30 on the SIAS indicate a clinical relevant level of social anxiety (Stangier et al., 1999). The German version of the SPS/SIAS has shown high levels of internal consistency and convergent, but deficient discriminant validity (Heinrichs et al., 2002).

#### Symptoms of depression

Depressive symptoms were measured using the German version of the Beck Depression Inventory (BDI-II; Hautzinger et al., 2006). The self-report measure consists of 21 items relating to symptoms of depression. The items are rated on a 4-point scale indicating the severity of symptoms and are rated for the past 2 weeks including today. Higher scores indicate more severe depressive symptoms. The BDI-II has shown good psychometric properties in clinical and non-clinical samples (Kühner et al., 2007).

#### General psychopathology

In order to measure psychopathology and psychological distress, the German version of the Brief Symptom Inventory (BSI; Derogatis and Melisaratos, 1983; Derogatis, 1993; Franke, 2000) was used. The BSI is a 53-item short form of the Symptom Check List 90 (SCL-90). It produces the same nine primary symptom dimensions (somatization, obsessive-compulsity, interpersonal sensitivity, depression, anxiety, hostility, phobic anxiety, paranoid ideation and psychoticism). Furthermore, three global indices measure general psychological distress. These include the Global Severity Index (GSI), the Positive Symptom Total (PST), and the Positive Symptom Distress Index (PSDI). Each item is rated on a 5-point Likert scale ranging from 0 (not at all) to 4 (extremely) and is considered to be rated for the experience of the past 7 days including today.

### Procedure

At the beginning of the study, participants provided written informed consent. The consent form stated that the purpose of the study was to “evaluate the relationship of mental visualization and psychological distress.” Subjects were informed that participation was voluntary, and that they could discontinue at any time. Following this, participants were invited to fill in a socio-demographic questionnaire as well as the study questionnaires. Afterwards, skin conductance and electrocardiogram (ECG) leads were positioned on participants with the assistance of the research assistants. To assess skin conductance, 9 mm electrodes were attached to the thenar and hypothenar surface of the nondominant hand. A layer of an isotonic electrolyte gel was placed on the electrodes to increase conduction. For the assessment of ECG signals, participants placed three disposable Ag/AgCl electrodes on the manubrium sterni, the lowest part of the sternum and the lowest left rib. Skin conductance and ECG were registered and digitized using a Varioport biosignal recording device (Becker Meditec, Karlsruhe, Germany), that was controlled by a Windows computer with Variograph software (Becker Meditec, Karlsuhe, Germany). Skin conductance and ECG were recorded simultaneously with a sampling rate of 512 Hz. Skin conductance signal was converted to microsiemens (μS) and ECG signal to beats per minute (bpm). Skin conductance was missing for one, and ECG for 4 participants due to error.

Baseline physiological activity (skin conductance and ECG) was assessed during a 3-min period of rest. During this period, participants were instructed to sit quietly and relax. Afterwards, participants were asked to fill in baseline assessments of affect. Next, participants were informed that to practice and test mental visualization, they would be playing a virtual ball-tossing game called “Cyberball” with what they believed to be two other players (Williams et al., 2000). In reality, these players were computer generated. Participants were instructed to mentally visualize (as vividly as possible) the scene throughout the game (“Imagine what the others look like. What sort of people are they? Where are you playing? Is it warm and sunny or cold and rainy?”). Shortly after the instruction, the experimenter received a staged phone call informing them that the other players were ready to start. Then the game began.

At the beginning of the game, the participants received the ball and were then required to indicate to whom they would like to throw the ball by clicking on the appropriate player icon. After receiving the ball twice, participants were randomly assigned to one of the experimental conditions. If assigned to the inclusion condition, participants received the ball for roughly one-third of the total throws. If assigned to the ostracism condition, participants were totally excluded from the game and did not receive the ball ever again. The game lasted for a total of 30 throws. Following the game, participants filled in the affect scales and the manipulation checks (see above).

After completing the questionnaires, participants were told that the experimenter would have to check recordings of the physiological signals of the other players and were instructed to stay on their chair and wait until the experimenter would return to remove the electrodes. At the end of a waiting period of 15 min, the experimenter returned and asked the participants to fill in the affect scales. Skin conductance and ECG were recorded continuously throughout the ball-tossing game and the waiting period.

Finally, participants were debriefed and had the chance to comment on the study and ask questions of the researchers. The study was approved by the Ethical Committee of the Department of Psychology of Bielefeld University.

### Data reduction and analyses

Physiological data were pre-processed and analyzed using MATLAB version 7.7 (2008b, The MathWorks, Natick, Massachusetts) with the toolboxes ANSLAB (Wilhelm and Peyk, 2005) and Ledalab (available under www.ledalab.de). R-waves in the ECG data were identified automatically by ANSLAB software (Wilhelm and Peyk, 2005) and converted to bpm. Additionally, a visual artifact inspection was conducted. Artifactual data points were manually replaced, non-recognized R-waves were edited and sections with high proportions of artifacts were not evaluated. Similarly, raw data of skin conductance were screened for implausible artifacts and manually edited. For further analyses, mean levels of skin conductance and heart rate, respectively, during baseline, the Cyberball game and the waiting period were used.

### Statistical analyses

All statistical analyses were carried out using the Statistical Package for the Social Sciences SPSS 20. At first, differences between conditions on manipulation checks and emotional reactions were evaluated using independent-sample *t*-tests. Additionally, a series of analyses of variance (ANOVAs) were conducted to evaluate effects of the inclusionary status on self-reported levels of needs. As it was assumed that socially rejected subjects would report more threatened fundamental needs as well as more negative emotional reactions, all analyses were conducted one-tailed. For analyses of immediate reactions to the Cyberball game on skin conductance, heart rate and ratings of positive and negative affect, 2 (condition: ostracism vs. inclusion) × 2 (time: baseline, Cyberball game) ANOVAs with repeated measurement on the second factor were conducted. In a second step, 2 (condition: ostracism vs. inclusion) × 2 (time: Cyberball game, waiting period) ANOVAs with repeated measurement on the second factor were conducted to analyze long-term effects of social rejection. Additionally, to trace heart rate changes in a more time-sensitive manner, the first 150 s of the Cyberball game period were split into 15 sections of 10 s each. An explorative 2 (condition: ostracism vs. inclusion) × 16 (time: first 10 s of baseline plus 15 Cyberball sections) analysis of variance (ANOVA) with repeated measurement on the second factor was conducted to evaluate effects of the inclusionary status on the trend of heart rate. Furthermore, explorative analyses were executed to detect potential gender-specific reactions toward social rejection. In rejected subjects, a 2 (gender: female vs. male) × 2 (time: baseline, Cyberball) ANOVA with repeated measurement on the second factor was conducted for skin conductance, heart rate and ratings of positive and negative affect. In addition, separate series of ANOVAs for female and male subjects were conducted to evaluate gender-specific effects of the inclusionary status on self-reported levels of needs and emotional reactions. For all analyses, the effect size η^2^, the 90% confidence interval (CI) of the estimated effect sizes and the observed power of analyses are reported. When necessary, Greenhouse–Geisser corrections were applied and original degrees of freedom together with Greeenhouse–Geisser ε are reported.

## Results

The sample consisted of 50 subjects (25 females, 50.0%), of which 25 individuals (50.0%) were assigned to the inclusion and 25 individuals (50.0%) to the ostracism condition. The average age was *M* = 24.04 (*SD* = 3.11). Table 1 presents participants' means on the assessments.

### Manipulation checks and confounding factors

There were two manipulation checks assessing inclusionary status. Subjects in the ostracism condition reported that they have received the ball less often during the game than subjects in the inclusion condition [*M* = 7.92% vs. 31.38%; *t*_(47)_ = 10.11, *p* < 0.001]. Additionally, participants in the ostracism condition reported that they felt significantly less included and more rejected than participants in the inclusion condition [*M* = 3.68 vs. *M* = 7.84; *t*_(48)_ = 8.47, *p* < 0.001]. After adjustment for multiple testing (*p* = 0.05/5 = 0.01), there were no differences between conditions on the assessments of confounding factors like imagination ability, vividness of imagination, subjective arousal during imagination, and familiarity to the situation (all *p*'s > 0.01; see Table 2).

**Table 2:** **Means and standard deviations (in parentheses) of fundamental needs, levels of emotional involvement, manipulation checks, and confounding factors**.

	**Ostracism (*n* = 25)**	**Inclusion (*n* = 25)**	***P***
**LEVEL OF FUNDAMENTAL NEEDS^a^^,^^d^**			
Belonging	13.24 (5.93)	28.04 (35.54)	0.023
Control	9.92 (5.05)	21.64 (16.64)	<0.001
Self-esteem	18.16 (6.95)	28.76 (35.49)	0.075
Meaningful existence	11.72 (6.02)	27.24 (35.71)	0.019
**LEVEL OF EMOTIONAL INVOLVEMENT^b^^,^^d^**			
Anxiety	1.50 (1.18)	1.32 (0.56)	0.248
Sadness	2.25 (1.70)	1.32 (0.69)	0.008
Anger	2.45 (1.74)	1.32 (0.69)	0.002
Guilt	1.33 (1.05)	1.36 (0.70)	0.458
Disgust	1.67 (1.37)	1.08 (0.28)	0.026
Shame	1.38 (0.92)	1.28 (0.68)	0.342
Happiness	3.25 (1.92)	4.56 (1.00)	0.002
**MANIPULATION CHECKS**			
What percent of throws did you receive?	7.92 (9.20)	31.38 (6.81)	<0.001
Included-excluded^c^	7.84 (1.72)	3.68 (1.75)	<0.001
**CONFOUNDING FACTORS^b^**			
How well were you able to visualize the scene?	4.25 (1.54)	5.12 (1.24)	0.034
Has your imagination been realistic?	4.04 (1.81)	4.84 (1.28)	0.083
How vivid was your imagination?	3.58 (1.67)	4.44 (1.33)	0.052
Have you been aroused while imagining the scene?	3.29 (1.33)	3.52 (1.19)	0.531
Has the imagined scene been familiar to you?	3.63 (1.95)	3.60 (1.71)	0.962

a *Each need score represents an average of three questions*.

b *Rated on a 7-point Likert scale*.

c *This was a 9-point Likert scale very included-very excluded as anchors*.

d *Analyses were conducted one-tailed*.

### Emotional reactions

All *t*-tests analyzing self-rated levels of emotions were adjusted for multiple testing (*p* = 0.05/7 = 0.007) and were conducted one-tailed, which is justified given the directionality of the predictions (see above). Ostracized subjects reported significant higher levels of anger, *t*_(47)_ = 3.03, *p* < 0.01, and lower levels of happiness, *t*_(47)_ = 3.02, *p* < 0.01 (see Table 2). No significant differences were found on other levels of emotional involvement (all *p*'s > 0.007; see Table 2).

### Self-reported level of needs

Analyses of self-reported levels of needs after the Cyberball game were all conducted one-tailed (see above). Participants that were excluded from game reported significant lower levels of Belonging, *F*_(1, 48)_ = 4.22, *p* < 0.05, partial η^2^ = 0.08, η^2^ 90% CI [0.00, 0.22], power = 0.54, Control, *F*_(1, 48)_ = 11.36, *p* < 0.001, partial η^2^ = 0.19, η^2^ 90% CI [0.05, 0.34], power = 0.92, and Meaningful Existence, *F*_(1, 48)_ = 4.59, *p* < 0.05, partial η^2^ = 0.09, η^2^ 90% CI [0.00, 0.23], power = 0.57. Ratings of Self-Esteem did not differ between conditions, *F*_(1, 48)_ = 2.15, *p* = 0.08, partial η^2^ = 0.04, η^2^ 90% CI [0.00, 0.16], power = 0.31 (see Table 2).

### Heart rate reactivity

**Figure 1:**
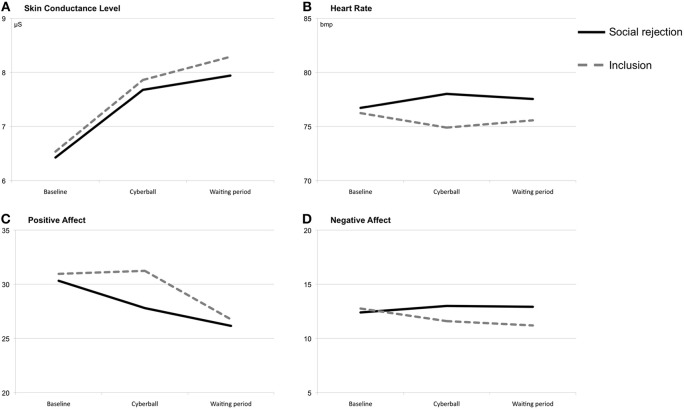
**Means at baseline, during/after the Cyberball game and during/after the waiting period**. Note: Total scores of the positive and negative affect scales range from 10 to 50. **(A)** Means of skin conductance level (at baseline, during/after the Cyberball game and during/after the waiting period). **(B)** Means of heart rate (at baseline, during/after the Cyberball game and during/after the waiting period). **(C)** Means of positive affect ratings (at baseline, during/after the Cyberball game and during/after the waiting period). **(D)** Means of negative affect ratings (at baseline, during/after the Cyberball game and during/after the waiting period).

For heart rate reactivity, an ANOVA with repeated measures showed a significant interaction effect of time × condition, *F*_(1, 44)_ = 5.66, *p* < 0.05, partial η^2^ = 0.11, η^2^ 90% CI [0.01, 0.25], power = 0.66, with increased heart rates during the Cyberball game in the ostracism condition and decreased rates in the inclusion condition (see Figure 1). No main effects were found for time, *F*_(1,44)_ < 0.01, *p* = 0.97, partial η^2^ < 0.01, η^2^ 90% CI [00, 0.00], power = 0.04, and condition, *F*_(1, 44)_ = 0.28, *p* = 0.60, partial η^2^ = 0.01, η^2^ 90% CI [0.00, 0.09], power = 0.08. For the waiting period, the ANOVA showed no significant effects [time: *F*_(1, 44)_ = 0.04, *p* = 0.84, partial η^2^ = 0.00, η^2^ 90% CI [0.00, 0.03], power = 0.05; condition: *F*_(1, 44)_ = 0.57, *p* = 0.46, partial η^2^ = 0.01, η^2^ 90% CI [0.00, 0.10], power = 0.12; time × condition: *F*_(1, 44)_ = 1.38, *p* = 0.25, partial η^2^ = 0.03, η^2^ 90% CI [0.00, 0.14], power = 0.22].

**Figure 2:**
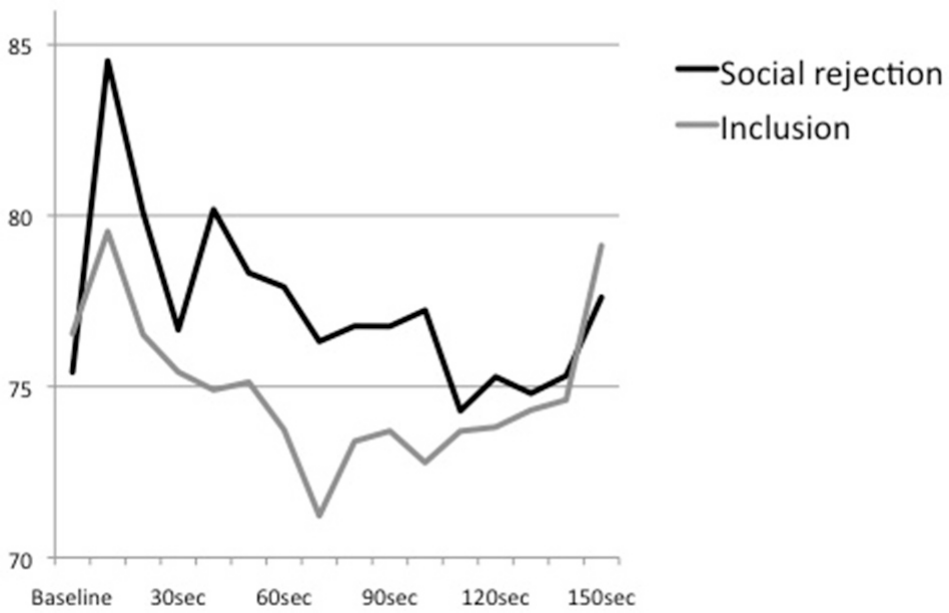
**Means of heart rate at baseline and the first 150 s of the Cyberball game (divided into sections of 10 s)**.

An additional ANOVA with repeated measures examining the trend of heart rate over the time course of the Cyberball game showed a significant main effect of time, *F*_(15, 675)_ = 6.41, *p* < 0.01, partial η^2^ = 0.13, η^2^ 90% CI [0.05, 0.18], power = 0.99, ε = 0.321. However, the interaction effect of time × condition, *F*_(15, 675)_ = 1.75, *p* = 0.13, partial η^2^ = 0.04, η^2^ 90% CI [0.00, 0.07], power = 0.60, ε = 0.321, and the main effect of condition, *F*_(1, 45)_ = 0.51, *p* = 0.48, partial η^2^ = 0.01, η^2^ 90% CI [0.00, 0.11], power = 0.11, were not significant (see Figure 2). All *post-hoc* analyses remained non-significant.

### Skin conductance level

The repeated-measures ANOVA of SCLs showed a significant main effect of time, *F*_(1, 47)_ = 41.70, *p* < 0.001, partial η^2^ = 0.47, η^2^ 90% CI [0.29, 0.59], power = 1.0 (see Figure 1). During the Cyberball game, both conditions showed heightened SCLs. Neither a significant main effect of condition, *F*_(1, 47)_ = 0.03, *p* = 0.86, partial η^2^ < 0.01, η^2^ 90% CI [0.00, 0.03], power = 0.05, nor a significant interaction effect of time × condition, *F*_(1, 47)_ = 0.04, *p* = 0.85, partial η^2^ < 0.01, η^2^ 90% CI [0.00, 0.03], power = 0.05, could be found. Similarly, a repeated-measures ANOVA revealed a significant main effect of time during the waiting period, *F*_(1, 47)_ = 8.74, *p* < 0.01, partial η^2^ = 0.16, η^2^ 90% CI [0.03, 0.31], power = 0.84 indicating increasing SCLs. Again, no significant effects were found for condition, *F*_(1, 47)_ = 0.11, *p* = 0.75, partial η^2^ < 0.01, η^2^ 90% CI [0.00, 0.07], power = 0.06, and the interaction of time × condition, *F*_(1, 47)_ = 0.49, *p* = 0.49, partial η^2^ = 0.01, η^2^ 90% CI [0.00, 0.10], power = 0.11.

### Positive affect

On the ratings of positive affect the ANOVA with repeated measures showed a significant interaction effect of time × condition, *F*_(1, 48)_ = 4.67, *p* < 0.05, partial η^2^ = 0.09, η^2^ 90% CI [0.00, 0.23], power = 0.58 (see Figure 1). Only subjects in the ostracism condition reported less positive affect immediately after the Cyberball game. Additional significant immediate effects could not be found [time: *F*_(1, 48)_ = 2.99, *p* = 0.09, partial η^2^ = 0.06, η^2^ 90% CI [0.00, 0.19], power = 0.41; condition: *F*_(1, 48)_ = 1.53, *p* = 0.22, partial η^2^ = 0.03, η^2^ 90% CI [0.00, 0.15], power = 0.24]. For the waiting period, the interaction of time × condition reached significance, *F*_(1, 48)_ = 4.70, *p* < 0.05, partial η^2^ = 0.09, η^2^ 90% CI [0.00, 0.23], power = 0.58. Included subjects showed a stronger decrease in positive affect during the waiting period than ostracized subjects. There was no significant main effect of condition, *F*_(1, 48)_ = 1.27, *p* = 0.27, partial η^2^ = 0.03, η^2^ 90% CI [0.00, 0.14], power = 0.20. Furthermore, a significant main effect of time was found, *F*_(1, 48)_ = 21.82, *p* < 0.001, partial η^2^ = 0.31, η^2^ 90% CI [0.14, 0.46], power = 0.99. Subject's ratings of positive effect decreased over time.

### Negative affect

A repeated-measures ANOVA showed a significant interaction effect of time × condition for the ratings of negative affect, *F*_(1, 48)_ = 6.51, *p* < 0.05, partial η^2^ = 0.12, η^2^ 90% CI [0.01, 0.26], power = 0.72, with increasing scores in the ostracism and decreasing scores in the inclusion condition (see Figure 1). Main effects of time, *F*_(1, 48)_ = 0.66, *p* = 0.42, partial η^2^ = 0.01, η^2^ 90% CI [0.00, 0.11], power = 0.13, and condition, *F*_(1, 48)_ = 0.88, *p* = 0.35, partial η^2^ = 0.02, η^2^ 90% CI [0.00, 0.12], power = 0.16, did not reach significance. For the waiting period of 15 min, the ANOVA showed neither a main effect of time, *F*_(1, 48)_ = 0.52, *p* = 0.48, partial η^2^ = 0.01, η^2^ 90% CI [0.00, 0.10], power = 0.11, nor an interaction effect of time × condition, *F*_(1, 48)_ = 0.23, *p* = 0.63, partial η^2^ = 0.01, η^2^ 90% CI [0.00, 0.08], power = 0.08. However, there was a significant main effect of condition with higher ratings on negative affect in the ostracism condition, *F*_(1, 48)_ = 6.14, *p* < 0.05, partial η^2^ = 0.11, η^2^ 90% CI [0.01, 0.26], power = 0.70.

### Explorative analyses of gender-specific reactions toward social rejection

In rejected subjects, a repeated-measures ANOVA of SCLs revealed a significant interaction effect of time × gender, *F*_(1,22)_ = 4.64, *p* < 0.05, partial η^2^ = 0.17, η^2^ 90% CI [0.00, 0.38], power = 0.58. Female subjects showed a larger increase in SCL than male subjects. Additionally, the ANOVA showed significant main effects of time, *F*_(1,22)_ = 25.07, *p* < 0.01, partial η^2^ = 0.53, η^2^ 90% CI [0.26, 0.67], power = 0.99, and gender, *F*_(1,22)_ = 4.46, *p* < 0.05, partial η^2^ = 0.17, η^2^ 90% CI [0.00, 0.38], power = 0.56. Furthermore, a repeated-measures ANOVA for ratings of negative affect showed a significant interaction effect of time × gender, *F*_(1, 23)_ = 4.29, *p* = 0.05, partial η^2^ = 0.16, η^2^ 90% CI [0.00, 0.36], power = 0.54, with increasing scores in female subjects and stable scores in male subjects after being rejected. All other repeated-measures ANOVAs did not reveal any significant effects (i.e., for heart rate, positive affect), all *p*'s > 0.05. Results of the ANOVAs evaluating gender-specific effects of the inclusionary status on self-reported levels of needs and emotional reactions are presented in Table 3.

**Table 3:** **Means and standard deviations (in parentheses) of fundamental needs and levels of emotional involvement separated by gender**.

	Female			Male		
	**Ostracism (*n* = 13)**	**Inclusion (*n* = 12)**	***P***	**Ostracism (*n* = 12)**	**Inclusion (*n* = 13)**	***P***
**LEVEL OF FUNDAMENTAL NEEDS^a^^,^^c^**						
Belonging	12.08 (5.93)	34.83 (51.51)	0.127	14.50 (5.81)	21.77 (2.28)	0.001
Control	9.08 (5.59)	21.64 (24.02)	0.044	10.83 (4.45)	19.77 (4.09)	<0.001
Self-esteem	15.77 (7.42)	35.67 (51.32)	0.179	20.75 (5.60)	22.38 (3.66)	0.402
Meaningful existence	10.38 (6.28)	34.75 (51.47)	0.103	13.17 (5.63)	20.31 (3.71)	0.001
**LEVEL OF EMOTIONAL INVOLVEMENT^b^^,^^c^**						
Anxiety	1.25 (0.62)	1.25 (0.45)	1.00	1.75 (1.54)	1.38 (0.65)	0.221
Sadness	2.58 (1.83)	1.33 (0.65)	0.018	1.92 (1.56)	1.31 (0.75)	0.111
Anger	2.50 (1.78)	1.33 (0.49)	0.024	2.42 (1.78)	1.31 (0.85)	0.028
Guilt	1.08 (0.29)	1.33 (0.65)	0.122	1.58 (1.44)	1.38 (0.76)	0.334
Disgust	2.00 (1.53)	1.17 (0.39)	0.046	1.33 (1.15)	1.00 (0.00)	0.170
Shame	1.42 (1.00)	1.50 (0.90)	0.416	1.33 (0.89)	1.08 (0.28)	0.167
Happiness	3.00 (1.81)	4.58 (1.08)	0.009	3.5 (2.07)	4.54 (0.97)	0.058

a *Each need score represents an average of three questions*.

b *Rated on a 7-point Likert scale*.

c *Analyses were conducted one-tailed*.

## Discussion

The present study aimed to explore the physiological reactions to social rejection. Participants played a virtual ball-tossing game in which they were either socially rejected or included. Results indicated that social rejection evoked an acceleration of heart rate while social rejection had no effect on subjects' SCLs. At the same time, the self-reported psychological consequences of social rejection documented the validity of the paradigm and of our application.

Social rejection as simulated by the Cyberball game elicits reactions of the ANS documented by an increase of heart rate in comparison to a non-excluded control condition. This finding corresponds to previous experiments that also found an increase of arousal caused by the exclusion condition in this paradigm. However, arousal response in these studies was limited to an increase of SCLs (Murray-Close, 2011; Shoulberg et al., 2011; Sijtsema et al., 2011; Kelly et al., 2012; Iffland et al., 2014), while in our study increases of SCL was found for excluded as well as for included subjects. A closer look at the previous studies shows that most experiments documenting an increase of electrodermal activity in response to social rejection did not provide the pattern of response for an included comparison group (Murray-Close, 2011; Shoulberg et al., 2011; Sijtsema et al., 2011; Iffland et al., 2014). Thus, it is plausible to assume that SCL reactions do not reflect a specific response to social rejection but result from an increase of participants' general level of activation/arousal while performing the task. In general, SCL is known to be sensitive to engagement of attention while heart rate reactions distinguish between emotional responses and task requirements (Frith and Allen, 1983). This may explain why heart rate but not a skin conduction response to rejection was found in this experiment.

However, our findings are inconsistent with a recent report that indicated a parasympathetic rather than sympathetic activation in response to social rejection (Gunther Moor et al., 2010) including a deceleration of heart rate. Several aspects may account for the opposite pattern of heart rate reactions to rejection. It may be speculated that subjects were emotionally more involved in the social-judgment task than in the Cyberball game since part of this experiment involves providing personal information and the intention is to make subjects believe that the rejection follows as a consequence of this information. Subjects were asked to send in photographs of themselves and were told that they would be judged by other participants. In contrast, subjects were only depicted as an animated figure in the Cyberball game and they did not provide any information about themselves to the co-players. Thus, it may be suggested that rejection is much more self-threatening and possibly more painful in the social-judgment task. As a consequence, the increasing activity of the dACC may finally result in the activation of the parasympathetic system (Thayer and Brosschot, 2005; Vogt, 2005). Including more personal information such as subjects' photographs, names, and attributes could enhance the ecological validity of the paradigm what may cause more emotional involvement. Ecological validity of the Cyberball version used in the current experiment is rather restricted. Another difference between both paradigms is the number of episodes of social rejection that were assessed. Whereas the present study was designed to examine only a single event of social rejection, subjects received rejecting feedback about 60 times in the social-judgment task. It is plausible to assume that such a series of repeated exposure to social rejection changes the stress reaction over the course of stimulations. Repeated episodes of social rejection may at some point turn into a blunted heart rate reaction. This idea is consistent with previous studies finding that subjects with a history of relational peer victimization showed blunted cortisol responses and heart rate reactions to social stressors (Ouellet-Morin et al., 2011a,b; Lovallo et al., 2012).

Moreover, time intervals that were analyzed differed enormously between tasks. While the present study used mean levels of heart rate during the whole Cyberball game which took approximately 3–5 min, Gunther Moor et al. (2010) analyzed initial heart rate reactions within an interval of 6 s. As it has been demonstrated that processing of social feedback may result in a phasic heart rate reaction (Somsen et al., 2000; Crone et al., 2003; van der Veen et al., 2004), it is plausible to assume that response to rejection may involve an immediate and transient decrease that is followed by an increase. This bi-phasic reaction would be consistent to the pattern of stress response to pictures of physical stress that also involves an initial orienting response characterized by a decrease of heart rate that is followed by an increase of arousal (Bradley and Lang, 2000; Levenston et al., 2000; Bradley et al., 2001). However, an additional, more time-sensitive analysis of our data did not reveal a bi-phasic reaction. Moreover, descriptive analyses indicated that heart rate of the rejected subjects increased around 30–40 s after starting the Cyberball game which is about the time point of being excluded from the game. In the course of the game, heart rate decreased and conditions converged. Thus, results suggest an initial acceleration of heart rate in reaction to social rejection which is followed by a stepwise decline. However, our study design and the results of the additional analysis are limited by the fact that the exact time point of being excluded from the game is not traceable in our data and time intervals were still larger than applied in prior studies. Hence, both paradigms, the social-judgment as well as the present Cyberball task, are not able to trace phasic changes appropriately as they do hardly allow to assess small and transient as well as more prolonged reactions simultaneously. More research using more appropriate paradigms and time intervals is needed to trace the time course of physiological reactions to social rejection, although valid social rejection paradigms that involve a direct social interaction are hard to standardize (Weik et al., 2010).

Other confounding context characteristics that have a likely effect on the central stress response were not controlled in the experiments and may account for the inconsistency of findings. This includes the effect of expectancy violation that seems to result in larger heart rate responses after rejection (Gunther Moor et al., 2010) as well as in the modulation of social pain (Ploghaus et al., 2003; Eisenberger and Lieberman, 2004). Unexpected and expected social rejection probably activate different (dorsal vs. rostral) regions of the ACC (Eisenberger and Lieberman, 2004) which in turn may result in a differing physiological response.

From a broader perspective it is obvious that it is overly simplistic to assume a single pattern of physiological reactions caused by social rejection and unaffected by task demands. In addition, it is likely that sample characteristics have an impact on the reactions to social rejection. For instance reaction patterns are modified by psychopathology and prior negative experiences (e.g., Borland et al., 2004; Zadro et al., 2006; Waldrip, 2007; Oaten et al., 2008; Gomez, 2009; Iffland et al., 2014). Moreover, it has recently been suggested that physiological reactions to social rejection may be gender-specific (Stroud et al., 2002; Weik et al., 2010). However, most studies used all-female samples or samples with predominantly women (Gunther Moor et al., 2010; Zöller et al., 2010; Murray-Close, 2011; Shoulberg et al., 2011; Sijtsema et al., 2011; Kelly et al., 2012) and did not consider potential gender effects at all. Although our study was not designed to examine gender effects, explorative analyses revealed a significant time × gender interaction for SCL in our sample. In line with Weik et al. (2010), women exhibited a greater reactivity toward social rejection. The analyses of self-reported psychological reactions confirmed a gender-specific response pattern. In women, rejection affected the variables of negative affect, control, sadness, anger, disgust, and happiness, while rejected men reported more anger and more threatened needs for belonging, control and meaningful existence. While these *ad-hoc* findings should be interpreted with caution, they still indicate that further studies on social rejection should consider gender-specific differences in explaining reactions to social rejection.

Consistent with prior studies (Williams et al., 2000; Leary et al., 2001; Buckley et al., 2004; Zadro et al., 2004; Gonsalkorale and Williams, 2007; Williams, 2007), social rejection caused negative psychological consequences. Immediately after the Cyberball game, rejected subjects showed more negative and less positive affect, reduced feelings of belonging, control and meaningful existence, and negative emotional reactions, such as anger and less happiness. However, while the effect of ostracism on the negative affect held up over the waiting period, ratings of positive affect of ostracized and included subjects did not differ in the long term. Decrease of positive affect during the waiting period in included subjects may be due to effects of uncertainty about the waiting situation without information about following tasks and duration of the waiting period, and boredom during the waiting period.

Our study has several limitations. The generalizability of our findings could be limited to relatively young subjects that are predominantly not in a relationship. Future studies should use much larger and more representative samples that allow the sample to be subdivided according to characteristics that are likely to be important. Our study aimed to examine physiological reactions to the Cyberball game. Although levels of skin conductance and heart rate indicate ANS and HPA axis reactivity, additional physiological and functional assessments (e.g., cortisol, RSA, fMRI) should be included to identify and evaluate varying patterns of effects of social rejection. Furthermore, the present study is limited by the use of mean levels of heart rate and SCLs, which comprised time intervals of 3–5 as well as 15 min. Further studies should include an iterative recording of diverse time intervals to examine phasic changes in heart rate as well as SCLs. Moreover, it should be considered whether existing social rejection paradigms are strong enough to elicit physiological reactions but also sufficiently standardized to trace phasic changes. Additionally, due to sample-size and restrictions of power potential effects of gender could not be analyzed in the present study. With respect to prior studies and the results of the explorative analyses presented in our study, further studies should address this limitation and emphasize the role of gender in reactions to social rejection.

## Conclusion

Social rejection evokes immediate physiological reactions. Although the effects of social rejection do not imply changes of SCLs, the ANS is affected by experiences of social exclusion indicating that behavior activation rather than inhibition is associated with socially threatening events. In addition, physiological reaction patterns might be influenced by inter-individual differences in life experiences, gender, and expectations of acceptance and rejection.

### Conflict of interest statement

The authors declare that the research was conducted in the absence of any commercial or financial relationships that could be construed as a potential conflict of interest.
